# The DPP-4 inhibitor sitagliptin improves glycaemic control and early-stage diabetic nephropathy in adolescents with type 1 diabetes using the MiniMed 780G advanced hybrid closed-loop system: a randomised controlled trial

**DOI:** 10.1007/s00125-024-06265-7

**Published:** 2024-09-14

**Authors:** Nancy S. Elbarbary, Eman A. Ismail, Manal H. El-Hamamsy, Marwa Z. Ibrahim, Amal A. Elkholy

**Affiliations:** 1 Department of Pediatrics, Faculty of Medicine, Ain Shams University, Cairo, Egypt; 2 Department of Clinical Pathology, Faculty of Medicine, Ain Shams University, Cairo, Egypt; 3 Department of Clinical Pharmacy, Faculty of Pharmacy, Ain Shams University, Cairo, Egypt; 4 Department of Clinical Pharmacy, Faculty of Pharmacy, Ahram Canadian University, Cairo, Egypt

**Keywords:** AHCL glucometrics, Diabetic nephropathy, Fasting lipids, MiniMed 780G, SDF-1, Sitagliptin, Type 1 diabetes

## Abstract

**Aims/hypothesis:**

Dipeptidyl peptidase-4 (DPP-4) inhibition has beneficial effects on various metabolic indicators in diabetes. Stromal cell-derived factor-1 (SDF-1) is expressed in diverse organs including the kidneys and is cleaved and inactivated by DPP-4 enzyme. The aim of this study was to conduct a randomised controlled trial to assess the effect of sitagliptin on diabetic nephropathy when used as an add-on therapy to the advanced hybrid closed-loop (AHCL) system in adolescents with type 1 diabetes and nephropathy.

**Methods:**

This open-label, parallel-group, randomised controlled trial took place at the Pediatric Diabetes Clinic, Ain Shams University, Egypt. Forty-six adolescents aged 14.13 ± 2.43 years on the MiniMed 780G system for at least 6 months before study, with HbA_1c_ ≤69 mmol/mol (8.5%) and diabetic nephropathy in the form of microalbuminuria, were randomly assigned to two groups (*n*=23 for each) based on a computer-generated randomisation sequence. The intervention group received oral sitagliptin 50 mg for 3 months. The other group used AHCL only and served as a control group. The primary outcome measure was the change in urinary albumin/creatinine ratio (UACR) after 3 months of administration of sitagliptin. The key secondary outcome measure was the change from baseline in SDF-1 levels after treatment.

**Results:**

Data for all participants were analysed. No significant difference was found between the groups as regards baseline clinical and laboratory characteristics as well as AHCL system settings (*p*>0.05). Serum SDF-1 levels were higher in all individuals with type 1 diabetes vs healthy control individuals (*p*<0.001). After 3 months, sitagliptin resulted in a significant decrease of SDF-1 levels from 3.58 ± 0.73 to 1.99 ± 0.76 ng/ml (*p*<0.001), together with improvement of UACR from 7.27 ± 2.41 to 1.32 ± 0.31 mg/mmol (*p*<0.001). In addition, sitagliptin reduced postprandial glucose, sensor glucose, coefficient of variation and total daily dose of insulin, while time in range 3.9–10.0 mmol/l (70–180 mg/dl) and insulin-to-carbohydrate ratio were significantly increased. Sitagliptin was safe and well-tolerated without severe hypoglycaemia or diabetic ketoacidosis.

**Conclusions/interpretation:**

Sitagliptin as an add-on therapy to AHCL had a reno-protective effect for individuals with type 1 diabetes and diabetic nephropathy, in addition to the improvement of time in range while reducing glycaemic variability and without compromising safety.

**Funding:**

This research received no specific grant from any funding agency in the public, commercial or not-for-profit sectors.

**Trial registration:**

ClinicalTrials.gov NCT06115460.

**Graphical abstract:**

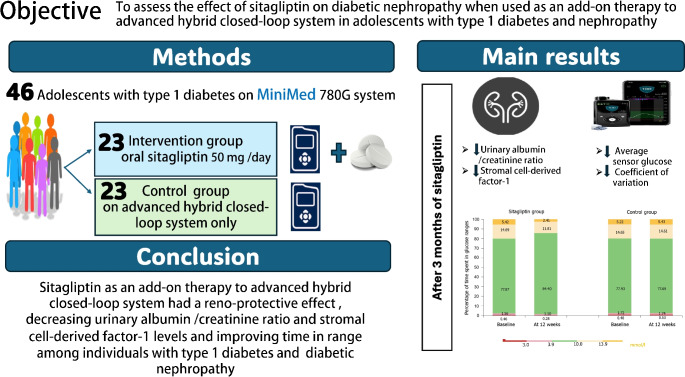


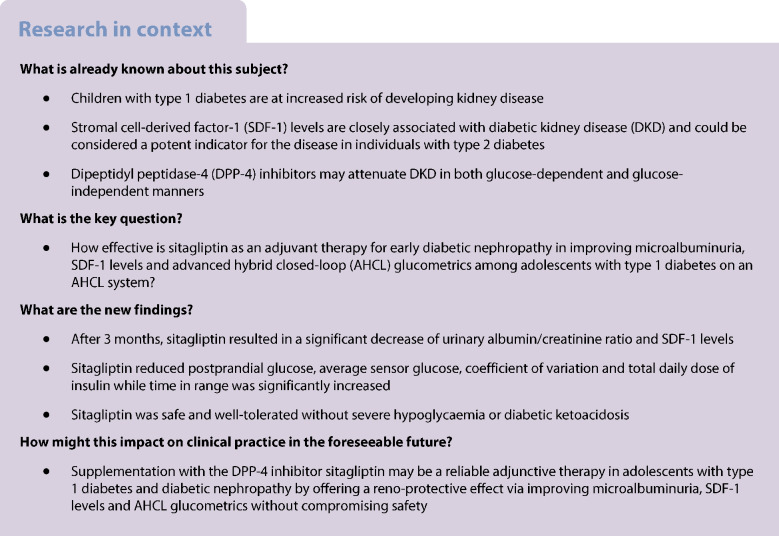


## Introduction

Children with type 1 diabetes mellitus are at increased risk of developing diabetic kidney disease (DKD). This may occur either due to acute kidney injury during diabetes onset that later progresses to chronic kidney disease or due to poor glycaemic control during the disease course causing chronic tubular damage that presents with DKD [1].

The incretin-based drugs glucagon-like peptide 1 (GLP-1) receptor agonists and dipeptidyl peptidase-4 (DPP-4) inhibitors, such as sitagliptin, are both used as glucose-lowering therapy for type 2 diabetes mellitus [2, 3]. DPP-4 inhibitors have a glycaemic lowering effect by different mechanisms including prevention of the degradation of both GLP-1 and glucose-dependent insulinotropic polypeptide (GIP) by DPP-4 enzyme. Therefore, DPP-4 inhibitors increase endogenous GLP-1 which suppresses glucagon secretion from alpha cells and inhibits hepatic glucose production, leading to glucose-lowering effects. Additionally, they slow gastric emptying and increase insulin sensitivity [4, 5].

Numerous clinical studies have shown the beneficial therapeutic effects of DPP-4 inhibitors in type 1 diabetes, where they decreased prandial insulin dose and total daily dose (TDD) of insulin [6, 7], inhibited glucagon secretion [8] and reduced blood glucose levels [9]. Moreover, DPP-4 inhibitors exert reno-protective effects in diabetic nephropathy through glucose-dependent [10, 11] and glucose-independent mechanisms [12–14].

Stromal cell-derived factor-1 (SDF-1), a member of the CXC chemokine family, is expressed in different organs and has multiple functions [15]. SDF-1 is cleaved and inactivated by the DPP-4 enzyme which is highly expressed in kidneys. DPP-4 inhibition leads to upregulation of renal SDF-1, causing multiple protective actions on the diabetic kidney through anti-inflammatory and anti-oxidative stress properties [16] and, consequently, amelioration of glomerular hypertension and hyperfiltration [17]. However, data on the role of SDF-1 in diabetic nephropathy are not sufficient [12]. Recently, serum SDF-1 levels were found to be a potent indicator for DKD in type 2 diabetes [15]. Being a mediator of neovascularisation, SDF-1 plays a critical role in progression of retinopathy [18] and diabetic neuropathy [19]. Increased levels of SDF-1 are also observed in individuals with an acute coronary syndrome conferring poor prognosis and risk of heart failure [20].

The introduction of the advanced hybrid closed-loop (AHCL [MiniMed 780G, Medtronic, Northridge, CA, USA]) system has provided major advances in improving glycaemic control and usability, with adjustable target glucose and automated correction boluses [21]. The current goal of insulin therapy in type 1 diabetes is to reach >70% of time in the target glucose range while minimising the burden of hypoglycaemia, together with decreasing the risk of development of long-term diabetes-related complications [22]. Sitagliptin showed an overall reduction in blood glucose concentrations in adults with type 1 diabetes on the AHCL system when used as an add-on therapy with insulin [23].

To our knowledge, no previous study has examined the role of sitagliptin in adolescents with diabetic nephropathy on the AHCL system. Therefore, this study was undertaken to investigate the role of sitagliptin as an adjuvant therapy for early diabetic nephropathy in adolescents with type 1 diabetes on the AHCL system and to assess its relation to microalbuminuria, SDF-1, lipid profile and AHCL glucometrics.

## Methods

This randomised controlled clinical trial included participants with type 1 diabetes defined according to the International Society for Pediatric and Adolescent Diabetes (ISPAD) guidelines [24]. Forty-six participants with type 1 diabetes and diabetic nephropathy aged from 11 to 18 years on the AHCL system were enrolled. Sex and ethnicity were self-reported. All the enrolled participants had the same race or ethnicity. Institutional review board (IRB) approval was obtained. Reporting of the study conforms to the Consolidated Standards of Reporting Trials 2010 statement [25]. The study protocol was approved by the Ethical Committee of Ain Shams University and registered at ClinicalTrials.gov (registration no. NCT06115460).

Inclusion criteria were individuals with type 1 diabetes on insulin pump therapy using the MiniMed 780G AHCL system for at least 6 months before study, with minimum insulin requirement of more than 8 units per day, HbA_1c_ ≤69 mmol/mol (8.5%) and diabetic nephropathy in the form of microalbuminuria (urinary albumin excretion [UAE] 3.5–35 mg/mmol). Persistent microalbuminuria was confirmed by abnormal results for two of three urine samples over a 3 to 6 month period prior to the study despite concomitant intake of angiotensin converting enzyme inhibitors (ACE-Is) [26].

Exclusion criteria were other diabetic complications than nephropathy, renal impairment due to causes other than diabetes, elevated liver enzymes, known allergy to sitagliptin, intake of oral hypoglycaemic medications 1 month before study and participation in a previous investigational drug study within the 3 months prior to the trial.

### Sample size

Sample size was calculated using PASS program version 15 (NCSs, Kaysville, UT, USA, 2017, ncss.com/software/pass), setting α error at 5% and power at 80%. Results from a previous study by Hattori [27] showed that urinary albumin/creatinine ratio (UACR) did not change in the 6 months before sitagliptin treatment (2.3 ± 19.9 mg/g creatinine) and decreased 6 months after sitagliptin treatment (−20.6 ± 24.6 mg/g creatinine). Based on this, the minimum sample needed was 17 cases per group. We included 23 participants in each group (with a total of 46 participants) to increase the power of the study and to take into consideration the dropout rate.

### Randomisation and study groups

**Fig. 1:**
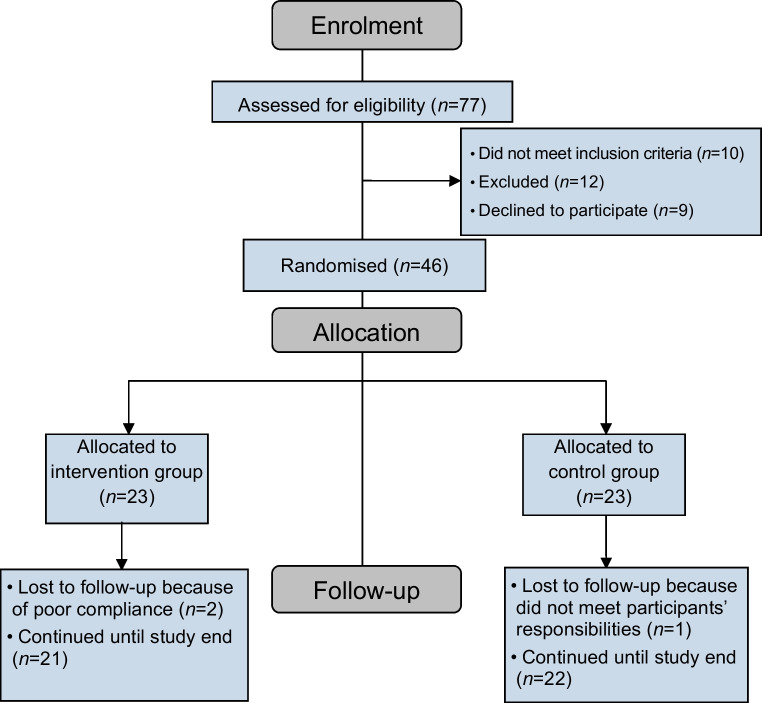
CONSORT flow diagram for the enrolled participants with type 1 diabetes and diabetic nephropathy on the MiniMed 780G system

A total of 77 individuals with type 1 diabetes and diabetic nephropathy using the MiniMed 780G AHCL system were screened for eligibility. Nine individuals declined to participate and 22 individuals did not meet inclusion/exclusion criteria; 12 of them were excluded before randomisation as they had one or more of the exclusion criteria: hepatitis C infection (*n*=6), autoimmune hepatitis (*n*=2), metformin intake (*n*=1) and participating in another clinical trial (*n*=3), while 46 participants were enrolled (Fig. 1).

Eligible participants were randomly assigned to one of the two groups by simple randomisation. Group I (sitagliptin group) consisted of 23 adolescent participants with diabetic nephropathy and using the MiniMed 780G AHCL system, who received sitagliptin in a dose of 50 mg (Januvia 50 mg film-coated tablets, marketing authorisation holder Merck Sharp & Dohme and manufactured by Organ Pharma, UK) orally once daily with the main meal with their insulin pump therapy for 3 months [28, 29]. Group II (control group) consisted of 23 participants who received their insulin via AHCL only without sitagliptin. Participants in both groups received an oral ACE-I captopril 25 mg tablet once daily (SmithKline Beecham, Egypt).

Data uploaded by MiniMed 780G AHCL users to CareLink Personal software over 3 months were analysed to identify demographic and system use characteristics. The mean glucose management indicator (GMI)-derived glycaemic metrics, which included the mean percentage of time in range (TIR) 3.9–10.0 mmol/l (70–180 mg/dl), time above range (TAR) >10.0 mmol/l (>180 mg/dl) and >13.9 mmol/l (>250 mg/dl), as well as time below range (TBR) <3.9 mmol/l (70 mg/dl) and <3.0 mmol/l (54 mg/dl), were determined for the overall 24 h day. In both groups, the mean sensor glucose (SG) levels, GMI, CV, insulin delivery patterns and insulin consumed in users with 14 or more days of SG data uploaded were assessed and reviewed every 2 weeks during the study period. Adjustments to the insulin/carbohydrate ratio (ICR) in both groups were performed whenever needed to optimise therapy and avoid acute complications among the enrolled participants during the monthly follow-up visits.

### Dietary intake

Together with pharmacological treatment, nutrient intake was tabulated from 24 h dietary recall, which was carried out by a dietitian through interview directly with the adolescents or caregivers. All participants were advised to follow the regular balanced diet schedule with optimal macronutrient distribution.

### Laboratory investigations

Fasting lipid profile and serum creatinine were measured using a Beckman Coulter AU480 chemistry analyser (Beckman Coulter Electronics, Hialeah, FL, USA). eGFR was calculated by the Chronic Kidney Disease Epidemiology Collaboration (CKD-EPI) equation. UACR was measured for all enrolled participants with microalbuminuria at the beginning of the study in an early-morning urine sample by an immuno-nephelometric method. Serum SDF-1 levels were measured using an ELISA kit from Bioassay Technology Laboratory (Shanghai, China) [15]. SDF-1 levels were compared with levels of 40 healthy control participants within the same age group as the patients and had a similar male to female ratio to obtain reference levels.

### Follow-up and endpoints

All participants were asked to announce meals, calculate carbohydrate amounts and pre-bolus before meals. All participants were closely and clinically followed up every 4 weeks for 3 months during the study period to monitor any potential adverse effects including gastrointestinal symptoms, upper respiratory tract infection and skin reactions. Additionally, metabolic events such as hypoglycaemia with all degrees of severity or diabetic ketoacidosis (DKA) and any adverse events that occurred during the study were recorded.

At the end of the 3 month period, participants were evaluated and UACR and SDF-1 levels were measured. The primary outcome measure was the effect on UACR after 12 weeks of follow-up of administration of sitagliptin. The secondary outcome measures included change in SDF-1 levels and improvement of TIR, average SG readings, CV and GMI. Further endpoints included the degree of insulin dose reduction during automated insulin delivery after sitagliptin supplementation.

### Statistical analysis

Data were collected, coded and entered into the Statistical Package for Social Science (IBM SPSS) version 27 (IBM Corporation, Chicago, IL, USA). Kolmogorov–Smirnov test was used to examine the normality of data. Comparison between sitagliptin and control groups as regards qualitative data was done using the χ^2^ test. The comparison between two independent groups with quantitative data and parametric distribution was done using an independent *t* test while non-parametric data were analysed using the Mann–Whitey test. To identify within-group changes (before and after 3 months of intervention), we applied paired-samples *t* tests for quantitative parametric data and the Wilcoxon test for non-parametric data. To compare the effects of sitagliptin between the intervention and control groups, ANCOVA adjusted for differences in baseline measures was performed. Pearson correlation coefficients were used to assess the correlation between SDF-1 and the studied variables. Multivariable linear regression analysis was used to assess the independent variables affecting UACR and SDF-1 levels. A *p* value <0.05 was considered significant in all analyses, which were conducted based on an intention-to-treat (ITT) principle, regardless of medication adherence.

## Results

### Baseline clinical characteristics of the study population

This study included type 1 diabetes 780G AHCL system users (*n*=46; 21 male and 25 female participants) who had ≥14 days of SG data on AHCL with a Guardian 3 (*n*=15) or Guardian 4 (*n*=31) sensor supplied by Medtronic, Northridge, CA, USA. Their ages ranged from 11 to 18 years with a mean age of 14.13 ± 2.43 years and mean diabetes duration of 6.96 ± 2.47 years. None of the participants were hypertensive or obese. At the study end, two participants in the sitagliptin group did not attend the follow-up visits and dropped out from the study. In the control group, one individual did not meet the participants’ responsibilities and dropped out (Fig. 1).

Upon comparison between baseline clinical and laboratory data among participants with type 1 diabetes and diabetic nephropathy using the MiniMed 780G AHCL system in sitagliptin and control groups, no significant difference was found, nor were there any significant differences in baseline MiniMed 780G AHCL system settings, usability or glucometrics between the groups (Tables 1 and 2; *p*>0.05).

**Table 1:** Comparison between sitagliptin and control groups as regards clinical and laboratory data at baseline and at the end of study among the enrolled participants with type 1 diabetes and diabetic nephropathy on the MiniMed 780G

Variable	Sitagliptin group				Control group				*p* value^b^
	Baseline (*n*=23)	At 3 months (*n*=21)	% Change	*p* value^a^	Baseline (*n*=23)	At 3 months (*n*=22)	% Change	*p* value^a^	
Age (years)	13.91 ± 2.31	–	–	–	14.35 ± 2.57	–	–	–	0.550^c^
Male, *n* (%)	13 (56.5)	–	–	–	8 (34.8)	–	–	–	0.139^d^
Disease duration (years)	7.39 ± 2.55	–	–	–	6.52 ± 2.35	–	–	–	0.236^c^
BMI SDS	0.98 (0.10, 1.76)	1.02 (0.11, 1.78)	5.17 (−1.04, 31.25)	0.069	1.11 (0.40, 1.60)	1.25 (0.40, 1.70)	6.25 (0.10, 25)	0.120	0.667
Systolic BP SDS	0.06 (−0.29, 0.68)	0.05 (−0.39, 0.58)	−13.64 (−16.67, −11.49)	<0.001	0.11 (−0.19, 0.70)	0.12 (−0.19, 0.26)	10.3 (1.10, 23.33)	0.231	0.032
Diastolic BP SDS	0.75 (0.67, 1.41)	0.65 (0.57, 1.01)	−20.83 (−21.74, −13.3)	<0.001	0.76 (0.65, 1.33)	0.86 (0.65, 1.10)	6.15 (0.01, 13.16)	0.293	0.038
Triglycerides (mmol/l)	0.73 ± 0.21	0.65 ± 0.15	−10.96 (−18.47, −2.35)	<0.001	0.74 ± 0.25	0.76 ± 0.23	2.70 (−1.85, 7.36)	0.214	<0.001
Total cholesterol (mmol/l)	3.96 ± 1.11	3.57 ± 1.16	−9.85 (−20.30, −3.65)	0.008	4.13 ± 0.93	4.14 ± 1.01	1.24 (0.35, 5.24)	0.439	<0.001
LDL-cholesterol (mmol/l)	1.91 ± 0.42	1.47 ± 0.35	−23.04 (−31.18, −11.78)	<0.001	1.97 ± 0.41	2.10 ± 0.38	4.68 (0.25, 11.28)	0.003	<0.001
HDL-cholesterol (mmol/l)	1.34 ± 0.34	1.67 ± 0.38	24.63 (8.14, 42.15)	<0.001	1.43 ± 0.32	1.41 ± 0.30	−1.40 (−5.67, 7.63)	0.617	<0.001
Serum creatinine (µmol/l)	62.78 ± 7.61	56.60 ± 7.23	−9.85 (−21.13, −2.36)	<0.001	60.13 ± 8.11	61.14 ± 5.35	0.09 (−0.10, 0.23)	0.767	0.021
eGFR (ml/min per 1.73 m^2^)	108.70 ± 6.28	119.43 ± 3.34	9.35 (8.77, 13.21)	<0.001	111.22 ± 6.01	110.96 ± 4.97	−0.23 (−0.90, 0.01)	0.299	<0.001
UACR (mg/mmol)	7.27 ± 2.41	1.32 ± 0.31	−81.84 (−98.41, −67.15)	<0.001	7.18 ± 2.11	8.42 ± 1.93	17.27 (3.65, 21.11)	<0.001	<0.001
SDF-1 (ng/ml)	3.58 ± 0.73	1.99 ± 0.76	−39.32 (−70.56, −32.10)	<0.001	3.53 ± 0.65	3.70 ± 0.72	4.25 (0.13, 5.98)	<0.001	<0.001

The data are shown as mean ± SD for parametric variables and as median (IQR) for non-parametric variables or as number and percentage for qualitative variables. The percentage change is expressed as median (IQR)

^a^*p* value was obtained from paired-samples *t* tests for parametric variables, Wilcoxon rank-sum test for non-parametric variables

^b^*p* value was obtained using ANCOVA unless specified

^c^Independent *t* test was used

^d^χ^2^ test was applied

*SDS* standard deviation score

**Table 2:** MiniMed 780G system performance between sitagliptin and control groups at baseline and at study end among the enrolled participants with type 1 diabetes and diabetic nephropathy

Variable	Sitagliptin group			Control group			*p* value^b^
	Baseline (*n*=23)	At 3 months (*n*=21)	*p* value^a^	Baseline (*n*=23)	At 3 months (*n*=22)	*p* value^a^	
Pre-meal glucose (mmol/l)	6.47 ± 0.92	6.55 ± 1.37	0.078	6.52 ± 1.2	6.57 ± 1.31	0.687	0.843
2hPPG (mmol/l)	8.23 ± 1.61	7.55 ± 1.34	<0.001	8.28 ± 1.57	8.31 ± 1.41	0.187	<0.001
Average SG (mmol/l)	8.42 ± 1.13	7.73 ± 0.96	<0.001	8.39 ± 1.03	8.37 ± 0.95	0.198	<0.001
GMI (eHbA_1c_ mmol/mol)	56.42 ± 2.39	50.78 ± 3.53	<0.001	54.61 ± 3.81	55.03 ± 2.94	0.625	<0.001
GMI (eHbA_1c_ %)	7.23 ± 0.14	6.80 ± 0.28	<0.001	7.13 ± 0.23	7.14 ± 0.14	0.677	<0.001
CV (%)	38.81 ± 1.29	34.11 ± 2.27	<0.001	38.39 ± 1.14	38.32 ± 1.18	0.299	<0.001
TIR 3.9–10 mmol/l (%)	77.87 ± 4.23	84.40 ± 5.15	<0.001	77.93 ± 4.30	77.69 ± 4.65	0.791	<0.001
TBR <3.9 mmol/l (%)	1.56 ± 0.41	1.10 ± 0.17	<0.001	1.72 ± 0.21	1.74 ± 0.19	0.898	<0.001
TBR <3.0 mmol/l (%)	0.46 ± 0.21	0.28 ± 0.1	0.001	0.48 ± 0.2	0.53 ± 0.22	0.467	<0.001
TAR 10.0–13.9 mmol/l (%)	14.69 ± 3.84	11.81 ± 2.87	<0.001	14.65 ± 3.91	14.61 ± 2.95	0.905	<0.001
TAR >13.9 mmol/l (%)	5.42 ± 1.33	2.41 ± 0.99	<0.001	5.22 ± 1.48	5.43 ± 1.41	0.946	<0.001
TDD (U/day)	51.82 ± 5.51	41.91 ± 4.63	<0.001	51.59 ± 5.19	51.54 ± 5.02	0.997	<0.001
Bolus amount (U/day)	35.81 ± 2.26	27.72 ± 2.15	<0.001	35.45 ± 2.80	35.41 ± 2.65	0.873	<0.001
Auto correction amount (day)	7.83 ± 1.13	5.95 ± 0.88	<0.001	7.79 ± 0.96	7.83 ± 0.85	0.641	<0.001
Auto basal/basal amount (day)	16.01 ± 2.84	14.19 ± 2.12	0.002	16.14 ± 2.28	16.13 ± 2.11	0.935	0.005
Carbohydrates (g/day)	182.87 ± 17.94	167.65 ± 9.21	<0.001	182.00 ± 19.54	179.30 ± 17.34	0.346	0.013
ICR (g)	6.84 ± 0.96	8.64 ± 0.88	<0.001	6.93 ± 1.08	6.84 ± 0.76	0.271	<0.001
Smart Guard/week Auto Mode	82.13 ± 2.45	83.44 ± 3.27	0.179	82.27 ± 2.46	82.06 ± 2.39	0.699	0.118
Sensor wear (%)	81.89 ± 2.71	83.10 ± 3.10	0.191	81.93 ± 2.83	81.67 ± 2.77	0.712	0.167
Exit from AHCL per participant (*n*/week)	1.38 ± 0.71	1.31 ± 0.62	0.911	1.32 ± 0.76	1.29 ± 0.69	0.917	0.343
BG calibration (*n*/day)	2.45 ± 0.32	2.44 ± 0.34	0.907	2.54 ± 0.41	2.53 ± 0.31	0.889	0.107
Set change (*n* of days)	3.26 ± 0.51	3.00 ± 0.38	0.080	3.07 ± 0.52	3.05 ± 0.57	0.383	0.738
Reservoir change (*n* of days)	3.23 ± 0.48	3.21 ± 0.25	0.865	3.12 ± 0.37	3.14 ± 0.33	0.517	0.392

The data are shown as mean ± SD

Smart Guard/week Auto Mode: percentage of time the Auto Mode is active per week where the basal insulin dose and the correction boluses are automatically calculated and delivered to regulate glucose levels to a target sensor glucose amount

^a^*p* value was obtained from paired-samples *t* tests

^b^*p* value was obtained using ANCOVA

*BG* blood glucose

### Effect of sitagliptin supplementation on kidney function and SDF-1 levels

SDF-1 levels were higher in all participants with type 1 diabetes and diabetic nephropathy compared with healthy control participants (3.56 ± 0.68 vs 1.34 ± 0.54 ng/ml; *p*<0.001). Comparison between laboratory data in participants with type 1 diabetes and diabetic nephropathy on the MiniMed 780G AHCL system who received sitagliptin at baseline and post therapy showed significant reduction in SDF-1 levels (from 3.58 ± 0.73 to 1.99 ± 0.76 ng/ml; *p*<0.001), serum creatinine (from 62.78 ± 7.61 to 56.60 ± 7.23 μmol/l; *p*<0.001) and UACR (from 7.27 ± 2.41 to 1.32 ± 0.31 mg/mmol; *p*<0.001) with improvement in eGFR (from 108.7 ± 6.28 to 119.43 ± 3.34 ml/min per 1.73 m^2^; *p*<0.001) after sitagliptin treatment (Table 1). On the other hand, UACR and SDF-1 levels were significantly increased after 3 months of follow-up among the control group.

### Effect of sitagliptin therapy on BMI, blood pressure and lipid profile

As shown in Table 1, participants with type 1 diabetes and diabetic nephropathy on the MiniMed 780G AHCL system who received sitagliptin for 3 months had significantly lower systolic and diastolic blood pressure, triglycerides, total cholesterol and LDL-cholesterol with higher levels of HDL-cholesterol post therapy compared with baseline levels (*p*<0.001) and with the control group at study end (*p*<0.05). However, no significant difference was observed as regards BMI after sitagliptin therapy and, also, no significant difference was found regarding blood pressure or lipid profile among the control group except for LDL-cholesterol which was increased at study end (*p*=0.002).

### Impact of sitagliptin on MiniMed 780G system performance and glycaemic control

After 3 months of sitagliptin, there was a significant decrease in average SG (from 8.42 ± 1.13 to 7.73 ± 0.96 mmol/l; *p*<0.001), GMI (estimated HbA_1c_ [eHbA_1c_] mmol/mol [%]) (from 56.42 ± 2.39 to 50.78 ± 3.53 mmol/mol [7.23 ± 0.14% to 6.80 ± 0.28%]; *p*<0.001) and CV (from 38.81 ± 1.29% to 34.11 ± 2.27%; *p*<0.001). Moreover, a significant improvement was found in 2 h postprandial glucose (2hPPG) from 8.23 ± 1.61 to 7.55 ± 1.34 mmol/l (*p*<0.001) after sitagliptin therapy compared with baseline levels, which was also significant compared with the control group (Table 2).

**Fig. 2:**
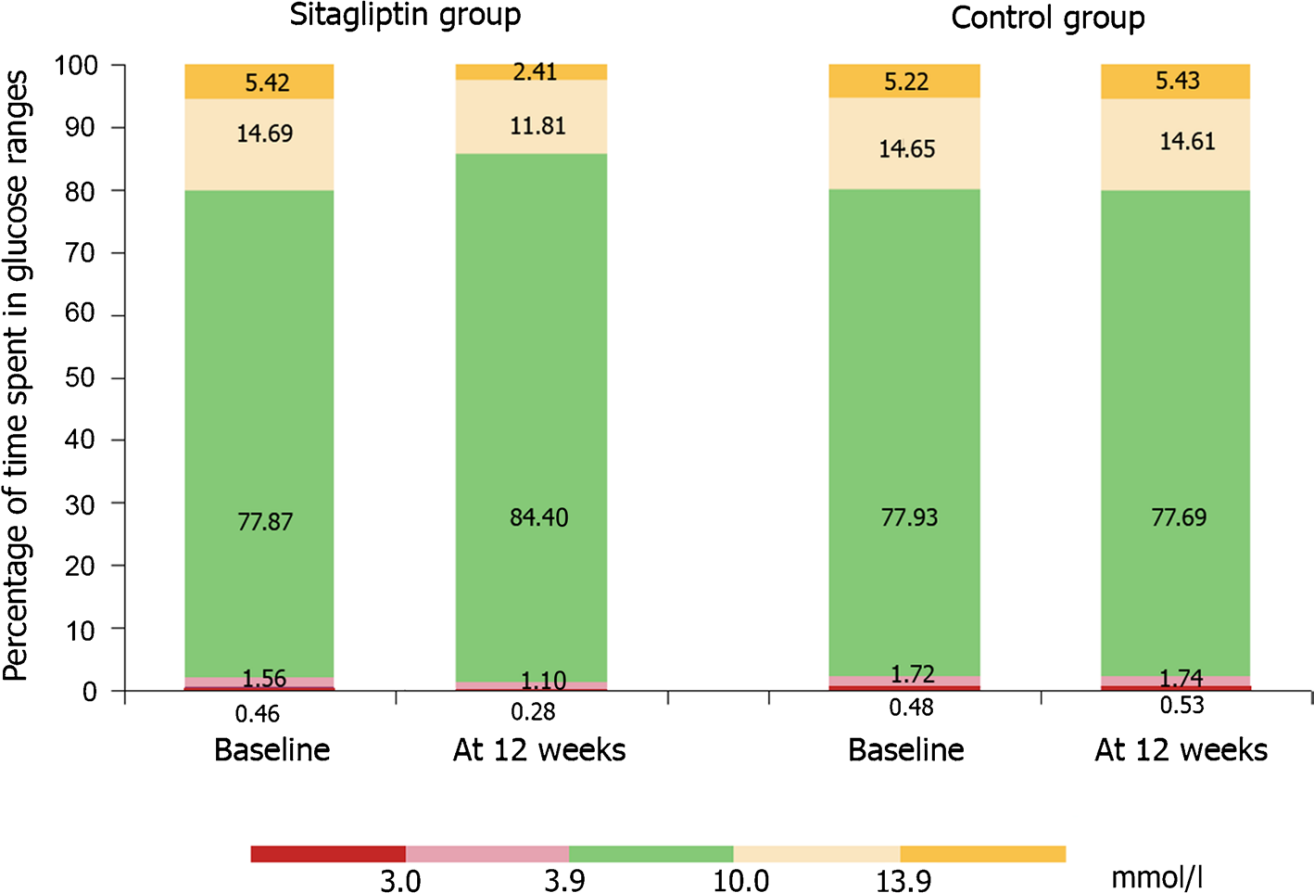
MiniMed 780G system performance showing the percentage of time spent in glucose ranges between sitagliptin and control groups at baseline and at study end among the enrolled participants with type 1 diabetes and diabetic nephropathy

Furthermore, improved glycaemic levels were observed among the intervention group as evidenced by the significant increase in TIR 3.9–10.0 mmol/l from 77.87 ± 4.23% to 84.40 ± 5.15%, together with a concomitant decrease in the level of hypoglycaemia; TBR <3.9 mmol/l was decreased from 1.56 ± 0.41% to 1.10 ± 0.17% and TBR <3.0 mmol/l was decreased from 0.46 ± 0.21% to 0.28 ± 0.1%, while TAR 10.0–13.9 mmol/l was decreased from 14.69 ± 3.84% to 11.81 ± 2.87% and TAR >13.9 mmol/l was decreased from 5.42 ± 1.33% to 2.41 ± 0.99% compared with baseline levels (*p*<0.001 for all; Fig. 2). The percentage change from baseline to study end between sitagliptin and control groups as regards MiniMed 780G glucometrics and glycaemic excursions also showed similar significant differences (Table 3). However, glucometrics did not change in control participants between baseline and study end (Table 2).

**Table 3:** Percentage change from baseline to study end between sitagliptin and control groups as regards MiniMed 780G glucometrics and glycaemic excursions among the enrolled participants with type 1 diabetes and diabetic nephropathy

Variable	Sitagliptin group	Control group	*p* value
Pre-meal glucose (mmol/l)	1.04 (0.85, 1.66)	0.87 (0.21, 1.83)	0.113
2hPPG (mmol/l)	−8.26 (−11.65, −4.38)	0.89 (0.30, 1.45)	<0.001
Average SG (mmol/l)	−8.19 (−12.65, −5.85)	−0.07 (−0.25, 0.28)	<0.001
GMI (eHbA_1c_ mmol/mol)	−10.01 (−11.80, −7.25)	0.02 (0.10, 1.54)	<0.001
GMI (eHbA_1c_ %)	−5.56 (−8.22, −4.05)	0.01 (0.09, 1.45)	<0.001
CV (%)	−12.47 (−15.67, −5.05)	−0.26 (−0.82, 0.10)	<0.001
TIR 3.9–10 mmol/l (%)	7.40 (6.41, 11.69)	−0.25 (−0.39, −0.02)	<0.001
TBR <3.9 mmol/l (%)	−29.41 (−50.04, 0.01)	0.01 (−0.03, 0.02)	<0.001
TBR <3.0 mmol/l (%)	−80.04 (−80.00, 0.01)	0.03 (−0.01, 0.06)	<0.001
TAR 10.0–13.9 mmol/l (%)	−20.01 (−29.49, −7.14)	0.06 (−0.01, 0.23)	<0.001
TAR >13.9 mmol/l (%)	−52.38 (−73.44, −17.50)	0.10 (0.01, 0.23)	<0.001
TDD (U/day)	−19.05 (−19.42, −12.61)	−0.07 (−0.39, 0.02)	<0.001
Bolus amount (U/day)	−21.05 (−29.07, −14.41)	−0.03 (−0.13, 0.19)	<0.001
Auto correction amount (day)	−14.71 (−34.07, −12.82)	0.10 (0.01, 0.22)	<0.001
Auto basal/basal amount (day)	−11.98 (−25.01, −2.00)	−0.60 (−1.35, −0.50)	0.005
Carbohydrates (g/day)	−5.88 (−10.64, −5.38)	−3.39 (−5.52, 0.03)	0.013
ICR (g)	19.05 (9.46, 40.35)	0.60 (0.03, 1.69)	<0.001

The data are shown as median (IQR)

*BG* blood glucose

### Carbohydrate intake, insulin delivery and ICR during study period

A significant reduction in carbohydrate intake was observed between sitagliptin and control groups at the study end (*p*=0.013). Among the sitagliptin group, carbohydrate intake declined from 182.87 ± 17.94 to 167.65 ± 9.21 g/day (*p*<0.001) while no significant difference was found in the control group (182.00 ± 19.54 vs 179.30 ± 17.34; *p*=0.346). This was reflected in reduction of TDD of insulin (from 51.82 ± 5.51 to 41.91 ± 4.63 U/day; *p*<0.001) and reduction of both bolus amount (from 35.81 ± 2.26 to 27.72 ± 2.15 U/day; *p*<0.001) and auto correction dose (from 7.83 ± 1.13 to 5.95 ± 0.88 per day; *p*<0.001) after 3 months of sitagliptin treatment. These changes were not observed in the control group. The ICR was significantly less aggressive and was adjusted to be 8.64 ± 0.88 g instead of 6.84 ± 0.96 g (*p*<0.001) after 3 months of sitagliptin treatment, reflecting lower bolus amount, while the ICR was slightly intensified in the control group and was adjusted to be 6.84 ± 0.76 g at study end instead of 6.93 ± 1.08 g but with no significant difference (*p*=0.271) (Table 2). It is worth mentioning that there was no need to modify sitagliptin dose to adjust glycaemic control during the follow-up visits.

### Relation between UACR and the studied variables among all participants with type 1 diabetes and nephropathy on the MiniMed 780G system

Baseline UACR levels were positively correlated to total cholesterol (*r*=0.418, *p*=0.003), SDF-1 (*r*=0.480, *p*=0.002), average SG (*r*=0.812, *p*<0.001) and CV (*r*=0.750, *p*<0.001) while UACR levels were negatively correlated to eGFR (*r*=−0.331, *p*=0.025) and TIR (*r*=−0.695, *p*<0.001). There were no correlations with other variables including blood pressure. Multivariable regression analysis showed that baseline SDF-1, average SG, CV and TIR were the significant independent variables that affected UACR among the studied participants with type 1 diabetes and diabetic nephropathy on the MiniMed 780G (Table 4).

**Table 4:** Multivariable regression analysis for independent variables affecting baseline UACR levels in all participants with type 1 diabetes and diabetic nephropathy on the MiniMed 780G

Independent variables	Unstandardised coefficients		Standardised coefficients	*p* value
	B	Standard error	β	
Total cholesterol (mmol/l)	0.021	0.086	0.022	0.811
eGFR (ml/min per 1.73 m^2^)	−0.294	0.146	−0.148	0.051
SDF-1 (ng/ml)	5.288	2.406	0.223	0.034
Average SG (mmol/l)	1.443	0.641	0.300	0.030
CV (%)	4.292	1.806	0.264	0.023
TIR 3.9–10 mmol/l (%)	−4.284	1.607	−0.242	0.011

When the analysis was repeated at the study end among the sitagliptin group to identify predictors of change of UACR, the percentage change of UACR levels was found to be positively correlated to SDF-1 (*r*=0.707, *p*<0.001), average SG (*r*=0.881, *p*<0.001) and CV (*r*=0.778, *p*<0.001) while UACR levels were negatively correlated to eGFR (*r*=−0.607, *p*=0.004) and TIR (*r*=−0.690, *p*<0.001). SDF-1, average SG and CV remained the significant independent variables in multivariable regression analysis (Table 5).

**Table 5:** Multivariable regression analysis for independent variables affecting the percentage of change of UACR levels among the sitagliptin group

Independent variables	Unstandardised coefficients		Standardised coefficients	*p* value
	B	Standard error	β	
(Constant)	−44.510	4.603		0.000
eGFR (ml/min per 1.73 m^2^)	−0.162	0.186	−0.113	0.395
SDF-1 (ng/ml)	0.116	0.048	0.231	0.028
Average SG (mmol/l)	2.740	0.957	0.416	0.012
CV (%)	0.649	0.166	0.411	0.001
TIR 3.9–10 mmol/l (%)	−0.144	0.448	−0.037	0.753

### Relation between SDF-1 levels and clinical and laboratory variables as well as AHCL system glucometrics

There were significant positive correlations between baseline SDF-1 and UACR (*r*=0.480, *p*=0.002), pre-meal glucose (*r*=0.739, *p*<0.001), 2hPPG (*r*=0.786, *p*<0.001), average SG (*r*=0.589, *p*<0.001) and TDD of insulin (*r*=0.339, *p*=0.021) while SDF-1 levels were negatively correlated to HDL-cholesterol (*r*=−0.452, *p*=0.005), eGFR (*r*=−0.641, *p*<0.001) and TIR (*r*=−0.519, *p*<0.001). Multivariable regression analysis showed that baseline eGFR, UACR, pre-meal glucose, 2hPPG, average SG and TIR were the significant independent variables that affected SDF-1 levels in all participants with type 1 diabetes and diabetic nephropathy on the MiniMed 780G (Table 6).

**Table 6:** Multivariable regression analysis for independent variables affecting baseline SDF-1 levels in all participants with type 1 diabetes and diabetic nephropathy on the MiniMed 780G

Independent variables	Unstandardised coefficients		Standardised coefficients	*p* value
	B	Standard error	β	
(Constant)	−27.623	4.458		<0.001
HDL-cholesterol (mmol/l)	−0.006	0.004	−0.099	0.164
eGFR (ml/min per 1.73 m^2^)	−0.049	0.015	−0.444	0.002
UACR (mg/mmol)	0.042	0.011	0.393	0.001
Pre-meal glucose (mmol/l)	0.139	0.031	0.826	<0.001
2hPPG (mmol/l)	0.127	0.029	1.125	<0.001
Average SG (mmol/l)	0.060	0.008	0.568	<0.001
TIR 3.9–10 mmol/l (%)	−0.218	0.043	−0.400	<0.001
TDD (U/day)	0.002	0.019	0.008	0.919

### Safety outcomes

There were no serious adverse events among either group and the full AHCL period was completed for all participants. Sitagliptin was safe and well-tolerated without reported episodes of severe hypoglycaemia or DKA. Skin irritations related to sensor use occurred in five participants and were resolved by local cream treatment. All participants in the intervention group were adherent to treatment and none of them discontinued the drug due to medication-related adverse events.

## Discussion

The highest DPP-4 enzyme levels are found in mammalian kidneys. Upregulation of DPP-4 in diabetic nephropathy makes it a promising potential therapeutic target [12]. SDF-1 is localised in podocytes and distal tubular cells of human kidneys [30] and is secreted under the effect of hyperglycaemia or ischaemic kidney injury [31]. However, although SDF-1 may ameliorate kidney injury, it promotes leukocyte infiltration and aggregation of inflammatory cells, together with potentiation of chemokines which contribute to a proliferative response in the kidney. All these factors ultimately lead to glomerular sclerosis, loss of podocytes, albuminuria and DKD [32].

In the current study, SDF-1 levels were higher in all the studied participants with type 1 diabetes and diabetic nephropathy vs healthy control participants. Baseline SDF-1 levels were positively correlated to blood glucose levels and UACR while SDF-1 was negatively correlated to TIR, HDL-cholesterol and eGFR. It has been reported that plasma SDF-1 levels were higher in type 2 diabetes than in normal control groups [33] and in mothers with gestational diabetes mellitus compared with normal pregnancy [34]. Renal biopsy revealed that SDF-1 levels were elevated in the kidneys of rodent models of diabetes and in individuals with DKD [35]. In line with our results, Lu et al [15] found that serum SDF-1 levels of individuals with type 2 diabetes and DKD were higher than in those without and were positively correlated to UACR and cystatin C levels while being inversely related to eGFR. SDF-1 was found to be an independent contributor to DKD [15] and also predicted eGFR decline over a year in individuals with coronary artery disease [36]. Furthermore, SDF-1 was identified as a risk factor for chronic kidney disease among individuals with diabetes [37].

In our study, DPP-4 inhibitor sitagliptin add-on therapy for 3 months resulted in a significant reduction of serum SDF-1 levels and UACR while eGFR was improved post therapy compared with baseline levels and with the control group. Our findings suggest that sitagliptin might have a reno-protective effect among individuals with type 1 diabetes on the MiniMed 780G AHCL system. It has been reported that the reno-protective effects of DPP-4 inhibitors could be due to increased half-life of their substrates such as GLP-1 and SDF-1a [12]. Of note, our participants were on an AHCL system and had relatively good glycaemic control. Both UACR and SDF-1 were positively correlated to blood glucose and the improvement in kidney parameters could be related to improved glycaemic outcomes; however, the possibility of a direct effect among those individuals cannot be excluded.

The literature has shown that DPP-4 inhibitors could improve two important risk factors for diabetic nephropathy, hyperglycaemia and albuminuria. This means there are potential beneficial effects on the kidney beyond glycaemic control [11]. Animal studies have shown that chronic low-dose sitagliptin administration was able to improve kidney function, decrease oxidative stress and abolish renal injury as it ameliorated glomerular, tubulointerstitial and vascular lesions in a rat model of type 2 diabetic nephropathy. The reno-protective effects of sitagliptin were most likely due to its ability to ameliorate hyperglycaemia and serum triglycerides [38].

Several studies have reported improvements in UACR with sitagliptin therapy, whether accompanied or not by changes in glycaemic control. However, the underlying mechanisms are far from being completely understood and clearly need further investigation [12, 39, 40]. Sitagliptin significantly decreased UACR in individuals with type 2 diabetes and micro- or macroalbuminuria over a 3 month study period [27, 41].

In another study [42], the change in UACR in individuals with type 2 diabetes and albuminuria who received sitagliptin was compared with those receiving sulfonylurea as an add-on to metformin. Both sitagliptin and sulfonylurea decreased albuminuria; this reduction was greater in the sitagliptin group and independent of glycaemic control [42].

Consistent with this observation, Mori and coworkers [43] showed that sitagliptin therapy for 6 months significantly reduced UACR in individuals with type 2 diabetes, especially those with high UACR at baseline, whereas individuals with normoalbuminuria (*n*=27), and also those with albuminuria (*n*=15), showed a reduction in UACR. However, glycaemic control was comparable in both groups. This suggests direct therapeutic effects of sitagliptin in early-stage diabetic nephropathy that independently influence blood pressure, body weight and glucose metabolism [43].

In this study, sitagliptin showed a weight-neutral effect, being an oral glucose-lowering agent with a low risk of hypoglycaemia, and no weight change was observed, which was mostly attributed to proper blood glucose control in the enrolled participants without hypoglycaemia. This is in agreement with other studies [44, 45].

Furthermore, sitagliptin add-on therapy resulted in a significant decrease of blood pressure as well as an improvement of lipid profile. A retrospective, observational study was conducted by Kubota et al [46] to identify the pleiotropic effects of sitagliptin, which demonstrated that sitagliptin not only seems to be effective in reducing blood glucose but also lowered blood pressure and improved fasting lipids. Sitagliptin causes a sodium diuretic effect due to increased GLP-1. Similar results were obtained by other studies where sitagliptin improved not only blood glucose levels but also body weight, blood pressure and lipids values in hyperlipidaemic individuals with type 2 diabetes [47, 48].

In addition, sitagliptin add-on therapy improved TIR and reduced postprandial glucose, average SG, CV and TDD of insulin, while ICR was significantly increased after 3 months, findings that were non-significant in the control group. Ellis et al [7] reported that DPP-4 inhibitors decreased HbA_1c_, blood glucose and insulin dosages in individuals with type 1 diabetes [7]. Underland et al [23] performed a randomised, double-blinded, placebo-controlled study to assess the role of 100 mg of sitagliptin as an add-on therapy in type 1 diabetes with an insulin-only closed-loop system for 25 h with timed meals. Sitagliptin reduced postprandial blood glucose and insulin delivery in the intervention group [23]. Similar results were found in individuals with type 2 diabetes [48].

In our study, both groups were followed up at the same intervals. However, further adjustments to the ICR in the intervention group (to be more relaxed and less intensive) helped to minimise the number of hypoglycaemic episodes and led to a reduction in insulin dose. Moreover, a reduction in carbohydrate intake/input was observed in our study between the sitagliptin and the control group. A possible reason for this could be due to the pharmacological effects of GLP-1, such as delayed gastric emptying [5, 49] promoting satiety [50], leading to a feeling of fullness after eating, which eventually decreased carbohydrate intake.

Notably, sitagliptin was safe and well-tolerated without reported severe hypoglycaemia or DKA. We found no significant difference between the control and treatment arms with regards to hypoglycaemia and no participants dropped out of the study due to hypoglycaemia. A meta-analysis that included only type 1 diabetes reported no serious adverse effects closely related to the DPP-4 inhibitors including ketoacidosis and pancreatitis [4].

Limitations of our study included the small number of enrolled participants. However, we believe that our study participants are representative of the larger population of interest within the geographical location. The absence of blinding of treating physicians or participants is considered another limitation. Moreover, longer study duration would provide better information on kidney function and SDF-1 levels in relation to diabetic nephropathy.

In conclusion, the DPP-4 inhibitor sitagliptin in a dose of 50 mg orally per day for 3 months was a safe add-on therapy to the AHCL system for adolescents with type 1 diabetes and diabetic nephropathy. Sitagliptin improved blood glucose levels and TIR while reducing glycaemic variability, insulin dose, UACR and SDF-1, resulting in a reno-protective effect among those participants. Further studies with longer follow-up periods of sitagliptin therapy are needed for verification of the results and examining their full efficacy and safety profiles, as well as investigating long-term effects on kidney disease progression and other diabetic complications. Whether sitagliptin improved diabetic nephropathy in type 1 diabetes through SDF-1 or a different mechanism represents an interesting area for future research.

## Abbreviations

ACE-I: Angiotensin converting enzyme inhibitor
AHCL: Advanced hybrid closed-loop
DKA: Diabetic ketoacidosis
DKD: Diabetic kidney disease
DPP-4: Dipeptidyl peptidase-4
eHbA_1c_: Estimated HbA_1c_
GLP-1: Glucagon-like peptide 1
GMI: Glucose management indicator
2hPPG: 2 h postprandial glucose
ICR: Insulin/carbohydrate ratio
ISPAD: International Society for Pediatric and Adolescent Diabetes
SDF-1: Stromal cell-derived factor-1
SG: Sensor glucose
TAR: Time above range
TBR: Time below range
TDD: Total daily dose
TIR: Time in range
UACR: Urinary albumin/creatinine ratio

## Declarations

### Data availability

The data are available on reasonable request from the authors.

### Authors’ relationships and activities

The authors declare that there are no relationships or activities that might bias, or be perceived to bias, their work.
